# The genetic diversity and structure of the Haflinger horse population in the Czech Republic

**DOI:** 10.5194/aab-67-323-2024

**Published:** 2024-07-04

**Authors:** Michaela Kulišťáková, Iva Jiskrová, Irena Vrtková, Petra Bartoňová, Tomáš Urban

**Affiliations:** 1 Department of Animal Breeding, Mendel University in Brno, 613 00 Brno, Czech Republic; 2 Department of Animal Morphology, Physiology and Genetics, Mendel University in Brno, 613 00 Brno, Czech Republic

## Abstract

The aim of the study was to describe the current state of genetic variability in the Haflinger breed in the Czech Republic using microsatellite markers, taking into consideration the numerous imports of breeding animals from abroad during the last 20 years and their impact on genetic diversity and population structure.

A total of 443 horses from five countries of origin (Austria – AUT, Germany – GER, Czech Republic – CZE, Italy – ITA, and Slovakia – SVK) bred in the Czech Republic were included in the study. A set of 16 microsatellite markers for parentage control from the International Society for Animal Genetics (ISAG) was used for genotyping. The total number of alleles in individual subpopulations ranged from 53 (SVK) to 117 (CZE). The mean number of alleles per locus was 6.69. Observed heterozygosity ( $H_{o}$ ) values ranged from 0.69 to 0.71 in all subpopulations. The most variable and informative locus (in terms of polymorphic information content – PIC) was VHL20, and the least variable was HTG6.

The $F_{\mathrm{is}}$ index was mostly negative or close to 0 for all populations and was -0.033 for the whole population. The overall $F_{\mathrm{st}}$ was 0.010, indicating a low level of differentiation between subpopulations. Cavalli-Sforza and Edwards chord genetic distances were low between the CZE, AUT, and GER populations, while the ITA and SVK populations were more distinct. The results of the discriminant analysis of principal components (DAPC) and the STRUCTURE analysis indicated a high degree of admixture among subpopulations. However, three to four genetic groups were clustered. The most distant populations were ITA and SVK, which we attribute to the low number of representatives in these subpopulations. A higher level of admixture due to gene flow was observed between the populations of GER, CZE, and AUT. Higher admixtures and the discovery of more distinct genetic clusters suggest that there is more significant gene flow from the countries of origin in the population of the Haflinger breed in the Czech Republic and that there is sufficient genetic variability and diversity to suggest sufficient opportunities for more intensive breeding.

## 1 Introduction

Haflinger (HFLG) horses have been bred in the Czech Republic since the Second World War. The main development in the breeding of Haflinger horses in the Czech Republic began after 1989, when horses began to be imported from abroad, mainly from Austria and Germany. Mass imports contributed greatly to the popularity of this breed, and the population of Haflinger horses is still growing (CSCHH, 2007).

Meanwhile, in Austria, breeding for modernization led to significant phenotypic changes, specifically an increase in withers, a reduction in robustness, the maintenance of long white veins, and improved trotting mechanics. Druml et al. (2016) also report that these strict selections of mares for elite stallions have led to a reduction in genetic diversity and a reduction in the breed's gene pool. In Italy, such a rigorous breeding process did not take place; thus, the original gene pool was preserved (Grilz-Seger et al., 2019).

Haflinger breeding in the Czech Republic has continued with an effort to get as close as possible to the appearance and character of horses coming from native countries; hence, in 2007, the Czech Haflinger Association joined the European Federation of Haflinger Horse Breeders. Subsequently, the Czech Haflinger Association became a member of the World Haflinger Breeding and Sports Federation in 2013 and, at the same time, the conditions for uniform breeding of this horse breed were agreed upon by member countries. During this period, many quality breeding horses began to be imported to the Czech Republic directly from Austria, and these still influence breeding today (CSCHH, 2017).

With the increasing globalization of animal breeding, the conservation and control of genetic diversity will become even more important (Groeneveld et al., 2010). While, originally, genetic diversity studies were conducted only from the analysis of pedigrees, in recent decades, this traditional method has been complemented or replaced by molecular data obtained from microsatellites (Engelsma et al., 2012) and, more recently, by genome-wide single-nucleotide polymorphism (SNP) markers (Petersen et al., 2013; Druml et al., 2018). In the Haflinger breed, genetic diversity has been studied mainly by means of pedigree analysis, but there are already studies based on microsatellite markers – for example, Grilz-Seger et al. (2019), Druml et al. (2016, 2018), and Sabbioni et al. (2007). Vostry et al. (2015) performed the initial study on the Czech population.

Sabbioni et al. (2007) analyzed the population structure of Italian Haflinger horses using pedigree analysis and calculated inbreeding coefficients, with an average inbreeding coefficient equal to 2.16 % ( ±0.05 ). Druml et al. (2016) evaluated the genetic variability of the Austrian Haflinger population using pedigree analysis. Their results showed an average inbreeding coefficient of 6.34. They also concluded that there has been an increase in inbreeding and a decrease in genetic variability, both of which are leading to a narrowing of the gene pool. They recommend the promotion of founder genomes in breeding, which are not currently used in breeding and therefore occur at low frequencies.

Grilz-Seger et al. (2019) investigated the effects of breeding at the genomic level. Their study showed that, in $F_{\mathrm{st}}$ and admixture analyses, the different subpopulations clearly differed from each other, with the South Tyrolean population showing the greatest distance ( $F_{\mathrm{st}}$ 7.1 %–7.3 %) from the others. Vostry et al. (2015), analyzing the Czech Haflinger population using 13 microsatellite markers, detected a total of 86 alleles. They concluded that the Czech Haflinger population is based on a smaller number of alleles, but this still seems to be sufficient, and the breeding program of the Czech Haflinger seems to be optimal.

The aims of this study were to investigate genetic diversity in the Haflinger population bred in Czech Republic, to calculate within-breed diversity, and to evaluate the genetic structure according to the countries of origin of the imported breeding animals using microsatellite markers.

## 2 Material and methods

### 2.1 Sample collection

The evaluation of the genetic diversity and structure of Haflinger populations in the Czech Republic was based on hair samples. A total of 443 individuals originating from the Czech Republic (CZE – 359), Austria (AUT – 59), Slovakia (SVK – 4), Italy (ITA – 4), and Germany (GER – 167) were included in the evaluation. The database includes individuals born from 1987 to 2021 in the proportion of 81 stallions, 285 mares, and 77 geldings. This enabled us to compare the five subpopulations' relative genetic relatedness both within and among subpopulations by country of origin.

### 2.2 Microsatellite markers

The set of 16 microsatellite markers recommended by the International Society for Animal Genetics (ISAG) – AHT4, AHT5, HMS1, HMS2, HMS3, HMS6, HMS7, HTG4, HTG6, HTG7, HTG10, VHL20, ASB2, ASB17, ASB23, and CA425 (ISAG/FAO Standing Committee, 2022) – was used for the analyses. The laboratory where analyses were conducted was regularly accredited according to the ISO/IEC 17025 standard and has been involved in the ISAG comparative testing.

### 2.3 Statistical analysis

Whole-population and within- and between-subpopulation diversity analyses were performed in R (v. 4.3.0; R Core Team, 2023). Basic statistics per locus were estimated by the hierfstat package, version 1.9.90 (Goudet and Jombart, 2022); these included observed heterozygosities ( $H_{o}$ ), within-population gene diversities ( $H_{s}$ ), overall gene diversities ( $H_{t}$ ), $D_{\mathrm{st}}$ , $H_{t^{\prime }}$ , $D_{{\mathrm{st}}^{\prime }}$ , $F_{\mathrm{st}}$ , $F_{{\mathrm{st}}^{\prime }}$ , observed $F_{\mathrm{is}}$ ( $F_{\mathrm{is}}$ ), and $D_{\mathrm{est}}$ (Nei, 1987; Jost, 2008). The fixation indices ( $F_{\mathrm{it}}$ , $F_{\mathrm{is}}$ , and $F_{\mathrm{st}}$ ) were obtained by Wright's F statistics (Weir and Cockerham, 1984) using the pegas R package, version 1.2 (Paradis, 2010).

The locus and the overall basic population parameters were calculated with the diveRsity package, version 1.9.90 (Keenan, 2013); these included A (the number of alleles), $A_{r}$ (the allelic richness), $H_{o}$ (the observed heterozygosity), $H_{e}$ (the expected heterozygosity), HWE (uncorrected p values from the chi-square test for goodness of fit to the Hardy–Weinberg equilibrium), $F_{\mathrm{is}}$ (the global $F_{\mathrm{is}}$ values observed per locus per population sample), and the mean values of parameters across loci per population. Polymorphic information content (PIC) per locus per population sample was calculated using Microsatellite Toolkit (Park, 2008).

Genetic differences between populations were evaluated by Cavalli-Sforza and Edwards chord distances ( $D_{\mathrm{ch}}$ ) (Takezaki and Nei, 1996) and pairwise $F_{\mathrm{st}}$ values following Weir and Cockerham (1984), implemented in the hierfstat R package, version 0.5-11 (Goudet and Jombart, 2022).

Population structure was evaluated using the Bayesian clustering approach (Pritchard et al., 2000) included in STRUCTURE 2.3.4. The number of presumptive clusters ( K ) was run from 2 to 6. The analysis was performed using Monte-Carlo-based replications with an admixture model and independent allele frequencies, using 10 ${}^{6}$ Markov chain Monte Carlo (MCMC) iterations with 10 ${}^{5}$ burn-in times. Ten replicate runs were performed for each value of K . The most likely K value and the log-likelihood coefficient (delta K ) in the result dataset were identified following Evanno et al. (2005) using the STRUCTURE HARVESTER tool (version 0.6.8) (Earl and von Holdt, 2012). To determine genetic structure and derive genetic admixture, discriminant analysis of principal components (DAPC) in the adegenet R package, version 2.1.10 (Jombart et al., 2010), was also used.

**Table 1:** Characteristic population differentiation (Nei, 1987; Jost, 2008) of 16 microsatellite loci analyzed in the Haflinger population in the Czech Republic ( n=443 ).

Loci	$H_{o}$	$H_{s}$	$H_{t}$	$H_{\mathrm{tp}}$	$D_{\mathrm{st}}$	$D_{\mathrm{stp}}$	$F_{\mathrm{st}}$	$F_{\mathrm{stp}}$	$F_{\mathrm{is}}$	$D_{\mathrm{est}}$
AHT4	0.746	0.727	0.735	0.737	0.007	0.010	0.011	0.013	-0.027	0.036
AHT5	0.742	0.6701	0.694	0.700	0.023	0.029	0.033	0.041	-0.106	0.088
HMS1	0.568	0.516	0.522	0.524	0.006	0.008	0.012	0.015	-0.101	0.017
HMS2	0.724	0.750	0.746	0.745	-0.004	-0.005	-0.006	-0.007	0.034	-0.020
HMS3	0.736	0.071	0.700	0.698	-0.009	-0.012	-0.013	-0.017	-0.038	-0.041
HMS6	0.605	0.655	0.643	0.641	-0.011	-0.014	-0.018	-0.022	0.077	-0.041
HMS7	0.635	0.736	0.735	0.735	-0.001	-0.001	-0.001	-0.001	0.137	-0.002
HTG4	0.692	0.620	0.614	0.613	-0.006	-0.007	-0.009	-0.012	-0.116	-0.019
HTG6	0.072	0.074	0.072	0.071	-0.002	-0.003	-0.029	-0.037	0.025	-0.003
HTG7	0.793	0.642	0.643	0.644	0.002	0.002	0.003	0.003	-0.235	0.006
HTG10	0.703	0.738	0.745	0.747	0.008	0.009	0.010	0.013	0.047	0.036
VHL20	0.803	0.846	0.855	0.857	0.009	0.011	0.010	0.012	0.052	0.069
ASB2	0.852	0.758	0.753	0.751	-0.005	-0.006	-0.007	-0.008	-0.125	-0.026
ASB17	0.783	0.838	0.838	0.838	0.001	0.001	0.001	0.001	0.066	0.004
ASB23	0.842	0.711	0.713	0.713	0.003	0.003	0.003	0.004	-0.185	0.009
CA425	0.720	0.751	0.732	0.727	-0.020	-0.025	-0.027	-0.034	0.042	-0.010
Overall	0.688	0.671	0.671	0.671	0.000	0.000	0.000	0.000	-0.026	-0.0001

The effective number of migrants per generation ( $N_{m}$ ) (Alcala et al., 2014) was estimated using the divMigrate function from the R package diveRsity (Keenan et al., 2013) to examine the direction and magnitude of migration between subpopulations.

## 3 Results

In the entire population of 443 individuals for the 16 microsatellite loci, the mean number of alleles per locus was 6.69 ( ±2.06 ). The overall observed heterozygosity was 0.693 ( ±0.006 ), and the expected heterozygosity (Nei, 1987) was 0.674 ( ±0.039 ). The results (Table 1) for the entire population show that each of the loci analyzed is polymorphic. The highest $H_{o}$ values were found for the markers ASB2 (0.852), ASB23 (0.842), and VHL20 (0.803). On the other hand, the lowest $H_{o}$ values were found for the HTG6 locus (0.072). The highest values of overall gene divergences ( $H_{t}$ ) were for markers VHL20 (0.855) and ASB17 (0.838), and the lowest values were for HTG6 (0.072). Low gene diversity ( $H_{o}$ and $H_{t}$ ) values for HTG6 markers were correlated with PIC values. The latter is lowest for the whole breed in HTG6 at 0.152 and is highest in VHL20 at 0.818. The lowest PIC value was recorded for the HMS1 locus at 0.429. All other loci had values higher than 0.6. The $F_{\mathrm{is}}$ index for the whole breed ranged from -0.235 to +0.137 . The genetic diversity ( $D_{\mathrm{st}}$ ) ranged from -0.025 to +0.030 , indicating small differences in heterozygosity between subpopulations. The other measures of differentiation ( $F_{\mathrm{st}}$ and $D_{\mathrm{est}}$ ) are also around zero.

The values of fixation index ( $F_{\mathrm{st}}$ ) according to Nei (1987) for individual loci range from -0.029 (HTG6) to +0.033 (AHT5). We consider these negative values to be zero. The $F_{\mathrm{st}}$ values are close to zero, indicating little differentiation among populations and thus higher genetic similarity among subpopulations divided by origin. Similar values for the $D_{\mathrm{est}}$ index (Jost, 2008) show a range of values for individual loci from -0.003 (HMS7) to 0.088 (AHT5). Thus, differentiation within populations is very low. The $F_{\mathrm{is}}$ index expressing the reduction in heterozygosity took values ranging from -0.235 (HTG7) to +0.137 (HMS7). The mean value was -0.026 , indicating a slight excess of heterozygotes.

Table 2 shows the values of the F statistics (Weir and Cockerham, 1984) for individual loci and the overall values within the entire Haflinger population in the Czech Republic. Fit index values such as the reduction in the heterozygosity of an individual due to non-random mating and population subdivision relative to the total population (overall inbreeding coefficient) were around zero ( -0.086 HMS3 to 0.058 HMS7), with an overall value of -0.024 . Index $F_{\mathrm{st}}$ represents the reduction in the heterozygosity of a subpopulation due to random genetic drift (also known as fixation index), and its values for individual loci were very low, close to zero ( -0.0001 HMS3 to 0.039 AHT5), and the overall $F_{\mathrm{st}}$ was 0.009. AHT5, HMS6, HTG1, ASB17, and CA425 microsatellites with $F_{\mathrm{st}}$ values higher than 0.01 had the most significant contribution. The values of $F_{\mathrm{is}}$ (reduction in heterozygosity of an individual due to nonrandom mating within its subpopulation) for individual loci were very low and mostly negative ( -0.086 HMS3 to 0.055 HMS7), and the overall $F_{\mathrm{is}}$ was -0.033 .

**Table 2:** F statistics (Weir and Cockerhan, 1984) of 16 microsatellite loci analyzed in the Haflinger population in the Czech Republic.

	$F_{\mathrm{it}}$	$F_{\mathrm{st}}$	$F_{\mathrm{is}}$
AHT4	0.001	0.005	-0.005
AHT5	-0.034	0.039	-0.076
HMS1	-0.039	0.003	-0.042
HMS2	-0.048	0.004	-0.052
HMS3	-0.086	-0.0001	-0.086
HMS6	-0.010	0.012	-0.022
HMS7	0.058	0.004	0.055
HTG4	0.034	0.010	0.024
HTG6	-0.046	0.006	-0.052
HTG7	-0.030	-0.001	-0.029
HTG10	-0.017	0.014	-0.031
VHL20	-0.005	0.003	-0.009
ASB2	-0.067	0.009	-0.077
ASB17	-0.021	0.025	-0.047
ASB23	-0.064	0.001	-0.065
CA425	-0.002	0.018	-0.020
Overall	-0.024	0.009	-0.033

**Table 3:** Genetic diversity in subpopulations. N is the average number of animals with the determined genotype for all loci, A is the total number of detected alleles, $A_{r}$ is the allelic richness, $H_{o}$ is the observed heterozygosity, $H_{e}$ is the expected heterozygosity, HWE is the Hardy–Weinberg equilibrium ( p value), and $F_{\mathrm{is}}$ is the coefficient of inbreeding.

Subpopulation	N	A	$A_{r}$	$H_{o}$	$H_{e}$	HWE	$F_{\mathrm{is}}$
AUT	54.88	101	3.40	0.71	0.68	0.999	-0.045
CZE	355.25	117	3.49	0.70	0.68	0.786	-0.035
GER	15.69	85	3.24	0.69	0.67	0.575	-0.027
ITA	4	65	3.24	0.72	0.64	0.000	-0.129
SVK	3.94	53	2.84	0.71	0.58	0.176	-0.233

Table 3 describes the rates of genetic diversity in each subpopulation. Allelic richness ( $A_{r}$ ) is a measure of genetic diversity indicative of a population's long-term potential for adaptability and persistence. The lowest value of $A_{r}$ of 2.84 was found in the ITA subpopulation, while the highest value was found in the CZE and AUT populations (3.49 and 3.40). The values of observed heterozygosity ( $H_{o}$ ) were high in all subpopulations (0.69–0.71). The values of expected heterozygosity ( $H_{e}$ ) were lower (0.58–0.68) than $H_{o}$ . The lowest $H_{e}$ value was in SVK. Individual subpopulations did not show deviations from HWE, only the ITA subpopulation deviated significantly from genetic equilibrium. The $F_{\mathrm{is}}$ index was negative for all subpopulations (from -0.233 to -0.027 ), indicating a sufficient proportion of heterozygosity across all populations. For conservation purposes it is suitable to concern ourselves with maintaining levels of genetic diversity that are as high as possible, both in terms of heterozygosity and allelic richness.

The evaluation of genetic diversity for each subpopulation is presented in Tables S1–S5 in the Supplement. The total number of alleles in each subpopulation ranged from 53 (SVK) to 117 (CZE). Total allele counts in the other populations were 101, (AUT), 85 (GER), and 65 (ITA). The HTG6 locus had the smallest number of alleles (one, two, and three) in the ITA, SVK, and GER populations, respectively. In the CZE and GER subpopulations, the number of alleles was five at this locus. The highest number of alleles was detected at the ASB17 locus in the CZE population, with 12 alleles. For individual populations, the allele counts ranged from 5 to 12 (CZE), 5 to 9 (AUT), 3 to 8 (GER), 2 to 6 (ITA), and 2 to 5 (SVK). The lowest $A_{r}$ in all populations was found for the HTG6 marker (1.0–1.7). The total $A_{r}$ was lowest in the SVK population (2.84). The actual and observed heterozygosity $H_{o}$ was lowest in all populations for the HTG6 marker (0.18 CZE, 0.17 AUT, 0.2 GER, and 0.25 SVK). In the ITA population, the HTG6 marker was monomorphic with a $H_{o}$ value of 0. The HMS6 marker also showed a low $H_{o}$ value of 0.25 for the SVK population. High values of observed heterozygosity were detected in the CZE population for the markers VHL20 (0.85), ASB17 (0.82), and ASB23 (0.81). In the AUT population, high $H_{o}$ values were found for the markers ASB17 0.89, AHT4 0.86, VHL20 0.84, ASB2 0.84, HTG10 0.81, ASB23 0.80, and HMS3 0.80. In the GER population, high $H_{o}$ values were found for the markers AHT4 (0.88), VHL20 (0.82), and CA425 (0.80). In the ITA population, $H_{o}$ equal to 1.00 was detected in the four markers AHT5, HTG4, HTG7, and ASB2. In the SVK population, $H_{o}$ values equal to 1.00 were found in markers HTG4, HTG7, ASB2, ASB17, and ASB23. High $H_{o}$ values for most markers indicate sufficient genetic variability in the populations. In the AUT population, all loci were in the HWE equilibrium. In the CZE population, statistical deviation from HWE was found only at the ASB23 locus. The other loci were in equilibrium (HWE). In the GER population, statistical deviation from HWE was found only at the HTG4 locus. Statistical deviation from HWE equilibrium was found in the SVK population at the HTG10 and VHL20 loci and in the ITA population at the AHT5, HMS2, and HTG10 loci. These results are again influenced by the small number of samples in the ITA and SVK populations.

The polymorphic information content (PIC) takes values from 0 to 1. The higher the value, the higher the variability, and the more suitable the locus is for assessing diversity. A value of 0 means that the locus is monomorphic, and a value of 1 means that it is highly informative, having many alleles with balanced frequencies. If the PIC value is greater than 0.75, the locus becomes more informative (Park, 2008). The polymorphic information content (PIC) of the HTG6 locus in the ITA and SVK populations (lowly ranked individuals) had a value of 0, indicating that the locus is monomorphic and not useful for diversity assessment in these populations. But even so, the HTG6 locus has the lowest PIC values in the other populations at 0.057 (AUT), 0.120 (GER), and 0.169 (CZE). The VHL20 locus was determined to be the most informative in all populations except SVK, where the ASB17 locus had the highest PIC value (0.746). Except for the ITA population, with a value of 0.786, PIC values in the other populations were greater than 0.81. The results in the individual populations correspond with those for the whole HFLG breed, where HGT6 has a PIC value of 0.152 and VHL20 has a PIC value of 0.818.

**Figure 1:**
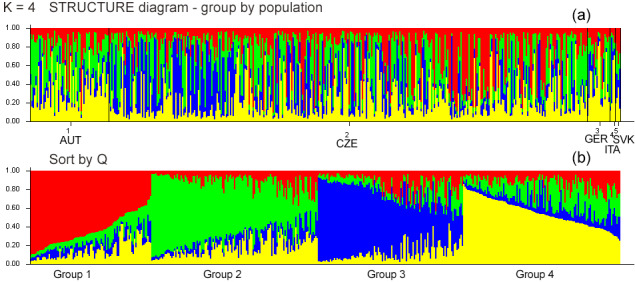
**(a)** Diagrams of the individuals from all five populations demonstrating assignment into different genetic clusters ( K ). Each individual is represented by a single column that is divided into segments whose size and color correspond to the relative proportion of the animal genome corresponding to a particular cluster. **(b)** K=4 based on ΔK , sorted by Q .

**Table 4:** $F_{\mathrm{st}}$ per pair following Weir and Cockerham (1984) (above diagonal) and Cavalli-Sforza and Edwards chord ( $D_{\mathrm{ch}}$ ) genetic distances (below diagonal) (Takezaki and Nei, 1996) among AUT, CZE, GER, ITA, and SVK populations.

	AUT	CZE	GER	ITA	SVK
AUT	–	0.012	0.004	-0.014	0.003
CZE	0.025	–	0.011	0.001	-0.002
GER	0.045	0.050	–	-0.013	-0.011
ITA	0.127	0.152	0.159	–	0.006
SVK	0.150	0.153	0.162	0.264	–

Pairwise $F_{\mathrm{st}}$ is a measure of the genetic differentiation between two populations based on the differences in allele frequencies between them. It is calculated as the ratio of the variance in allele frequencies between populations to the total variance in allele frequencies (Kitada et al., 2021). Pairwise $F_{\mathrm{st}}$ values among the five populations are presented in Table 4 and varied between -0.014 and 0.012. Negative values are considered to be zero and indicate complete panmixia. The $F_{\mathrm{st}}$ value indicates low genetic differentiation if it lies in the range of 0–0.05. The Cavalli-Sforza and Edwards chord ( $D_{\mathrm{ch}}$ ) were calculated to retrieve the relation among subpopulations (Table 4). The largest genetic distance was found between the ITA and SVK populations ( $D_{\mathrm{ch}}=0.264$ ). Both populations also differed from the others (AUT, CZE, GER) (0.127–0.162). However, these results may be influenced by the very low numbers of individuals in the ITA and SVK populations, which may not be representative samples. On the other hand, the lowest genetic distance was observed between the AUT, CZE, and GER populations (0.025–0.050), indicating a high level of admixture of populations by origin due to gene flow.

Bayesian clustering methods were used to identify genetic structures in the dataset. The five experimental groups formed distinct clusters when analyzed with STRUCTURE (Fig. 1). When all data were analyzed together, the optimal number of distinct genetic populations was based on the mean LnP( K ) =-14207.71 and ΔK=30.918 . The results suggested that the optimum population structure was at K=4 . Figure 1 and Table 5 show that the initial populations by origin do not correspond to the inferred clusters. Assignment proportions are relatively evenly distributed. In all four inferred clusters, individuals from the CZE and SVK subpopulations are equally distributed. Individuals from the AUT subpopulation are most represented in clusters 2 and 4, and from the GER and ITA subpopulations, they are most represented in cluster 2.

**Figure 2:**
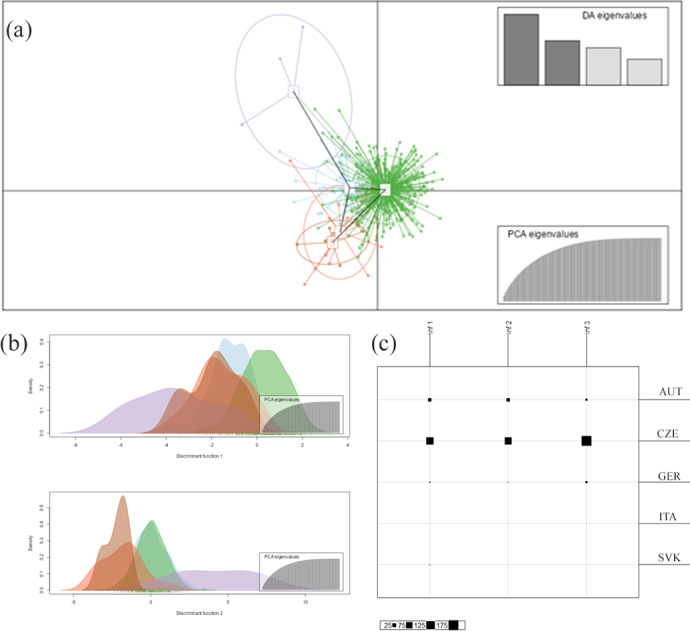
Inference of the subpopulations by DAPC analysis grouping Haflinger horses in the Czech Republic from different countries of origin together. **(a)** The axes represent the first two linear discriminants (LDs). Each circle represents a cluster, and each dot represents an individual. Numbers represent the different subpopulations identified by DAPC analysis (1-blue: AUT, 2-green: CZE, 3-orange: GER, 4-violet: ITA, 5-red: SVK); **(b)** discrimination functions 1 and 2; **(c)** the Bayesian information criterion (BIC) statistic results referring to differentiation between inferred and original clusters (AUT, CZE, GER, ITA, and SVK).

**Table 5:** Proportion of membership of each pre-defined population in each of the four clusters based on the STRUCTURE analysis at K=4 (the largest assignment proportion for each population is shown in bold).

	Inferred clusters				
Population	1	2	3	4	Number of
					individuals
AUT	0.138	**0.353**	0.183	**0.326**	59
CZE	**0.252**	0.238	**0.244**	0.266	359
GER	0.145	**0.401**	0.186	0.268	17
ITA	0.227	**0.426**	0.089	0.258	4
SVK	**0.268**	0.241	**0.260**	0.231	4

The discriminant analysis of the principal components (DAPC) was applied as a second approach to analyze the population structure and admixture. According to the graphical outputs by scatterplots (Fig. 2a, b), the original five subpopulations are clustered into three groups ( k=3 ). The distribution of individuals according to the Bayesian information criterion (BIC) analysis showed that inferred clusters do not correspond to actual groups. As expected, a certain level of admixture was revealed in all studied populations (Fig. 2c). The demonstrated marked admixture between subpopulations confirms the results of the STRUCTURE analysis. The agreement between prior and posterior assignment was 98.42 %.

Table 6 presents estimates of the effective number of migrants per generation ( $N_{m}$ ). The results show high migration rates from AUT to CZE (1000) and from GER to CZE (0.630). The migration rates from the small populations of ITA and SVK to CZE are much lower (0.288 and 0.289, respectively).

**Table 6:** Estimation of the number of migrants per generation ( $N_{m}$ ) between subpopulations.

	AUT	CZE	GER	ITA	SVK
AUT		1.000	0.587	0.133	0.112
CZE	0.926		0.501	0.114	0.102
GER	0.624	0.630		0.134	0.103
ITA	0.308	0.288	0.178		0.064
SVK	0.299	0.289	0.206	0.061	

## 4 Discussion

The aim of this study was to analyze genetic variability using microsatellite markers in the Haflinger breed originating from different countries and currently bred in the Czech Republic. The results were processed for the entire HFLG breed population bred in the Czech Republic and were further separately assessed for subpopulations according to the country of origin. We were interested in whether individual subpopulations bred and imported into the Czech Republic from different countries of origin are distinguishable from each other and whether or not increasing heterozygosity occurs in Czech breeding. Neumayr (2016) states that, in Haflinger horses, since their origin dates to a single founding stallion 249 Folie, breeding was based on inbreeding. If consanguineous breeding continued, the degree of undesirable homozygosity would increase.

A total of 443 individuals from five different countries of origin (CZE, GER, AUT, SVK, ITA) were included in the study. The total number of alleles analyzed by us for 16 microsatellites ranged from 53 (SVK) to 117 (CZE) in individual subpopulations. The low number of alleles in the SVK population may be due to the low number of individuals. In the ITA and GER populations with very small numbers of individuals, the number of alleles was higher (66 and 85) than in the SVK population. All analyzed microsatellite loci were polymorphic.

The average number of alleles per locus was 6.69 ( ±2.06 ). Vostry et al. (2015) evaluated the genetic diversity of the Haflinger breed using 369 random samples of horses obtained between 2000 and 2012 and found a total of 86 alleles for 13 microsatellites, with an average number of alleles per locus of 8.25. Almarzook et al. (2022) report similar results for the Arabian horse breed, with an average number of alleles per locus ranging from 6.33 to 7.58. Kusza et al. (2013) found 130 alleles, with an average number of 7.65 alleles per locus in the Hucul horse breed. There may be many reasons for the different number of alleles per locus, such as differences in the size of the studied population, differences in the number of microsatellites included in the study, or the breeding history of individual animal groups.

The observed heterozygosity for the entire population was 0.693 ( ±0.006 ) and the expected heterozygosity was 0.674 ( ±0.039 ). Putnova et al. (2019) report very similar values for the HFLG breed with $H_{o}$ 0.689 ( ±0.007 ) and $H_{e}$ 0.685 (0.037). Vostry et al. (2015) found similar but slightly lower values, reporting observed heterozygosity across microsatellite loci of 0.656. Here, we can observe a gradual increase in $H_{e}$ and $H_{o}$ from the oldest to the newest results for this breed in the Czech Republic, which may be due to gene flow from other countries in recent years. Biggi and Perrotta (2012) report $H_{o}=0.77$ and $H_{e}=0.73$ for one of the HFLG populations they studied. Jiskrova et al. (2016) found the opposite trend in the Czech population of Akhal-Teke horses – observed heterozygosity with a value of 0.648 was lower than expected heterozygosity with a value of 0.731, even though most Akhal-Teke horses were imported into the Czech Republic and were originally unrelated. Inbreeding is already evident. Similarly, Kusza et al. (2013) report a higher value of $H_{e}$ ( 0.747±0.099 ) than of $H_{o}$ ( 0.706±0.138 ), indicating a narrow genetic base in the Hucul breed. It seems that this trend does not yet occur in Haflinger horses; therefore, inbreeding does not occur. Druml et al. (2016) state that, although Haflinger horses are internationally bred, previous studies have shown that both genetic diversity and gene pool variability in the core breeding population of Austria are decreasing, while the average coefficient of inbreeding has increased from 6.3 % in the 1980s to 11.9 % for horses born after 2000.

When observing variability for individual loci, the highest $H_{o}$ values were found at the ASB2 (0.852), ASB23 (0.842), and VHL20 (0.803) loci. Conversely, the lowest $H_{o}$ values were found at the HTG6 locus (0.072). Vostry et al. (2015) has similar results for HFLG as we do (lowest $H_{o}$ of 0.217 at HTG6, highest $H_{o}$ if 0.844 at VHL20). Similarly, Putnova et al. (2019) report the lowest $H_{o}$ for HFLG at HTG6 at 0.130 and the highest at VHL20 at 0.810. As in our study, it has high $H_{o}$ values for markers ASB17 (0.800), ASB23 (0.808), and ASB2 (0.790). Vostra-Vydrova et al. (2018) found similar results for three cold-blooded horse breeds – the HTG6 microsatellite had the lowest value of $H_{o}=0.456$ , and the AHT4 microsatellite had the highest value of 0.810, followed by VHL20 with a value of 0.798. Czernekova et al. (2012) found the lowest values for the HTG6 microsatellite locus with $H_{o}$ 0.374 and $H_{e}$ 0.406 in the Kladruber horse breed. Then, like our study, the highest $H_{o}$ values were achieved by the microsatellites VHL20 (0.765), AHT4 (0.827), and ASB2 (0.821). The HTG6 locus is also the least variable and the VHL20 locus is the most variable in Hucul and Polish primitive horse breeds (Mackowski et al., 2015).

The highest values of total genetic divergence ( $H_{t}$ ) were found for the markers VHL20 (0.855) and ASB17 (0.838), and the lowest was found for HTG6 (0.072). Vostry et al. (2015) also reported the highest genetic diversity values for the microsatellite VHL20 (0.872) and the lowest for the microsatellite HTG6 (0.228). Low values of gene diversity ( $H_{o}$ and $H_{t}$ ) for the HTG6 marker correlated with PIC values. It is lowest for the entire breed at HTG6, with a value of 0.152, and it is highest at VHL20, with a value of 0.818. The lowest PIC value was recorded at the HMS1 locus at 0.429. All other loci had values higher than 0.6. Vostry et al. (2015) have a PIC value below 0.6 in their results for the microsatellites HTG6 (0.208), HMS1 (0.475), HTG4 (0.535), and HTG10 (0.560). In cold-blooded horses, Vostra-Vydrova et al. (2018) report a PIC value below 0.6 for the HTG6 (0.422) and HMS1 (0.521) loci. Putnova et al. (2019) report an overall PIC value of 0.639 for the HFLG breed.

The relatively low values of the F statistics ( $F_{\mathrm{it}}$ , $F_{\mathrm{st}}$ , and $F_{\mathrm{is}}$ ; Weir and Cockerhan, 1984) for individual loci and overall values ( -0.024 , 0.009, and -0.033 , respectively) were close to zero. The estimated $F_{\mathrm{is}}$ by Nei (1987) had similar values. There is no reduction in heterozygosity in the Haflinger population because of non-random mating relative to the total population and in subpopulations or because of random genetic drift. Similar results were also obtained by Vostry et al. (2015) for the entire HFLG population studied; the $F_{\mathrm{is}}$ ranged from -0.054 to 0.057, with an average value of -0.013±0.031 . This increased $F_{\mathrm{is}}$ value, according to them, corresponds to the fact that HFLG breeding in the Czech Republic is open to gene flow from other Haflinger populations. Putnova et al. (2019) report a $F_{\mathrm{is}}$ value of -0.005 for the HFLG breed, and Biggi and Perrotta (2012) report a value of -0.04 for the same breed. For cold-blooded horse breeds, $F_{\mathrm{is}}$ values range from -0.053 to 0.076 (Vostra-Vydrova et al., 2018). Kuzsa et al. (2012) found an average $F_{\mathrm{is}}$ value of -0.128 for Hucul horses, and for individual microsatellite loci, it ranged from 0.448 to -0.276 . Grilz-Seger et al. (2019) state that the genetic distance expressed by the $F_{\mathrm{st}}$ value was highest between South Tyrolean, Slovenian, and Austrian Haflinger horses (7.3 %–7.1 %).

The genetic diversity among the HFLG subpopulations may have been partly influenced by the low number of individuals, especially in the SVK and ITA subpopulations. There are differences between subpopulations, although small. The smallest number of alleles and the lowest allelic richness were in the SVK population, whereas the CZE population was the most variable. However, this was not reflected in the observed heterozygosity, which was balanced in all subpopulations. Only the $H_{e}$ value was lower in the SVK population. Nevertheless, only the ITA population was not in HWE. The $F_{\mathrm{is}}$ coefficients were negative in all subpopulations, but the lowest values were found in the ITA and SVK populations, which had the fewest individuals evaluated.

The lowest number of alleles was detected for the HTG6 locus in the ITA, SVK, and GER populations. The largest number of alleles (12) was detected in the CZE population at the ASB17 locus. The HTG6 locus had the lowest heterozygosity ( $H_{o}$ and $H_{e}$ ) and PIC values in all populations. Similarly, low variability at the HTG6 locus was found by Jiskrova et al. (2016) in populations of the Akhal-Teke breed bred in different countries.

High $H_{o}$ values for most markers indicate sufficient genetic variability in the HFLG subpopulations. All loci were in HWE balance only in the AUT population. In the CZE population, statistical deviation from HWE was detected only at the ASB23 locus; in the GER population, it was detected only at the HTG4 locus; in the SVK population, it was detected only at the HTG10 and VHL20 loci; and in the ITA population, it was detected at the AHT5, HMS2, and HTG10 loci. These results are again influenced by the small number of samples in the ITA and SVK populations. The VHL20 locus had the highest PIC values in all populations, except for SVK, where the ASB17 locus had the highest value (0.746). The results in each population are consistent with those for the entire HFLG breed, where HGT6 has the lowest PIC value (0.152) and VHL20 had the highest (0.818).

The low values of pairwise $F_{\mathrm{st}}$ and of Cavalli-Sforza and Edwards chord genetic distances in terms of genetic differentiation among subpopulations indicate considerable gene flow or mixing of populations by origin. These values also suggest that subpopulations are not sufficiently differentiated and may have a common history and breeding practices. The genetic distance between the populations appeared to be the highest between the ITA and SVK populations (0.264). Both populations also differed from the other (AUT, CZE, GER) (0.127–0.162). In turn, the small number of animals in these subpopulations may have influenced these results. Thus, the results are consistent with the relationship of individuals from different origins.

Druml et al. (2018) demonstrated a clear separation of the Austrian and Italian Haflinger clusters by genome-wide SNP marker analysis.

On the other hand, the lowest genetic distance was found between the AUT, CZE, and GER populations (0.025–0.050), indicating a high level of admixture. The high Nm values also indicate the high migration rates between the AUT, GER, and CZE subpopulations. Here, we expected a certain degree of admixture because, for breeding of the breed, crossbreeding of original Czech offspring (not conforming to the world standard) with stallions imported from autochthonous countries is used. The offspring of these stallions are already included in our research in the CZE subpopulation. Despite this, the CZE subpopulation is still distinguishable from the others, and the influence of the original Czech breeding based originally on the import of poor-quality and unusable horses from other countries, especially from Austria and Germany after 1989, can be seen.

Comparison of DAPC and STRUCTURE analysis results shows that the Haflinger population in the Czech Republic can be structured into a hypothetical three to four subpopulations according to the origin of imported breeding animals. However, both results confirm that the structure of the HFLG breed in the Czech Republic was not sufficiently differentiated and that there was a high level of admixture between subpopulations by origin. The subpopulations with the highest admixture were CZE, AUT, and GER. The greater diversity was caused by more limited gene flow from ITA and SVK. According to the discriminant analysis, individuals from each of the original subpopulations were found in all proposed populations in different proportions. The division of the HFLG breed into subpopulations according to the origin of breeding material does not completely determine the genetic differentiation within the HFLG breed bred in the Czech Republic.

## 5 Conclusions

This study examined the genetic structure and diversity in the population of Haflinger breed horses bred in the Czech Republic. Since the Haflinger breed is not native to the Czech Republic, we compared the degree of genetic connectivity between breeding animals imported from surrounding countries (AUT, GER, ITA, SVK) and horses born in the Czech Republic (CZE). When assessing the entire selected population of Haflinger horses, we found that the values of $H_{o}$ and $H_{e}$ were increased compared to previous research, indicating a constant gene flow from breeding populations in other countries. The results of genetic diversity for the entire population almost matched the results for individual subpopulations based on origin. The observed heterozygosity values were high in all subpopulations, which was confirmed by the low and mostly negative $F_{\mathrm{is}}$ index. This indicates sufficient genetic variability in all subpopulations. From the DAPC and STRUCTURE results, it is evident that, despite the differentiation of three to four genetic subpopulations, the breed has a low level of differentiation, and there is a high degree of admixture among all compared subpopulations. The populations of CZE, AUT, and GER appeared to be the least different. This may be due to the persistent popularity of purchasing breeding horses from abroad, especially from Austria, and the subsequent impact of these animals on Czech breeding. Given the high proportion of imported horses, there is currently no risk of inbreeding in the Czech Republic. On the contrary, sufficient genetic variability and diversity, gene flow, and distinguishable genetic groups in the Haflinger breed in the Czech Republic offer great opportunities for breeding and approaching internationally desired breed standards.

## Supplement

10.5194/aab-67-323-2024-supplement The supplement related to this article is available online at: https://doi.org/10.5194/aab-67-323-2024-supplement.

## Data Availability

The data used and analyzed during this study are available from the corresponding author upon reasonable request.
