# Supplementary material for: The genetic diversity and structure of the Haflinger horse population in the Czech Republic
Source: Arch Anim Breed. 2024 Jul 4;67(3):323–33. doi: 10.5194/aab-67-323-2024 (PMC13170701; doi:10.5194/aab-67-323-2024)
Supplement: The supplement related to this article is available online at: https://doi.org/10.5194/aab-67-323-2024-supplement. [file aab-67-323-2024-supplement.pdf]

Supplement of Arch. Anim. Breed., 67, 323–333, 2024
https://doi.org/10.5194/aab-67-323-2024-supplement
© Author(s) 2024. CC BY 4.0 License.

Open Access
Archives Animal Breeding

Supplement of

The genetic diversity and structure of the Haflinger horse population in the Czech Republic

Michaela Kulišťáková et al.

Correspondence to: Tomáš Urban (urban@mendelu.cz)

The copyright of individual parts of the supplement might differ from the article licence.

Table S1. Diversity characteristics of individual microsatellites in Austrian subpopulation

AUT	N	A	Ar	H_o	H_e	HWE	F_{is}	PIC
**AHT4**	59	7	3.79	0.86	0.75	0.144	-0.157	0.699
**AHT5**	58	7	4.17	0.79	0.77	0.495	-0.030	0.717
**HMS1**	49	5	2.4	0.65	0.57	0.841	-0.153	0.472
**HMS2**	58	7	3.57	0.71	0.71	0.990	0.003	0.652
**HMS3**	59	6	3.81	0.80	0.71	0.968	-0.129	0.649
**HMS6**	59	5	3.06	0.71	0.65	0.803	-0.090	0.574
**HMS7**	59	5	3.22	0.58	0.70	0.663	0.171	0.620
**HTG4**	58	6	3.04	0.59	0.65	0.122	0.101	0.546
**HTG6**	58	5	1.59	0.17	0.16	1.000	-0.070	0.057
**HTG7**	58	5	3.22	0.67	0.66	0.991	-0.022	0.586
**HTG10**	58	7	3.88	0.81	0.75	0.661	-0.076	0.695
**VHL20**	58	9	4.78	0.84	0.84	1.000	-0.003	0.817
**ASB2**	55	6	3.47	0.84	0.73	0.694	-0.152	0.667
**ASB17**	46	9	4.04	0.89	0.81	0.917	-0.105	0.781
**ASB23**	46	7	3.51	0.80	0.74	0.992	-0.087	0.645
**CA425**	40	5	2.82	0.65	0.70	0.979	0.070	0.646

Table S2. Diversity characteristics of individual microsatellites in Czech subpopulation

CZE	N	A	Ar	H_o	H_e	HWE	F_{is}	PIC
**AHT4**	359	6	3.82	0.74	0.75	0.795	0.021	0.711
**AHT5**	358	8	3.75	0.79	0.73	0.670	-0.083	0.680
**HMS1**	355	5	2.23	0.54	0.52	0.369	-0.026	0.419
**HMS2**	359	8	3.72	0.78	0.73	0.634	-0.073	0.685
**HMS3**	359	9	4.02	0.79	0.72	0.361	-0.093	0.675
**HMS6**	359	5	3.37	0.70	0.69	0.232	-0.017	0.633
**HMS7**	359	7	3.46	0.70	0.72	1.000	0.028	0.664
**HTG4**	359	6	3.00	0.61	0.62	0.905	0.020	0.554
**HTG6**	359	5	1.70	0.18	0.17	0.917	-0.055	0.168
**HTG7**	359	5	3.10	0.63	0.61	0.987	-0.021	0.560
**HTG10**	359	8	3.63	0.72	0.70	0.959	-0.035	0.640
**VHL20**	359	9	4.74	0.85	0.84	0.998	-0.017	0.814
**ASB2**	355	8	3.66	0.79	0.73	0.528	-0.074	0.682
**ASB17**	342	12	4.24	0.82	0.78	0.456	-0.053	0.755
**ASB23**	342	11	4.00	0.81	0.77	0.001	-0.059	0.721
**CA425**	342	5	3.37	0.73	0.71	0.989	-0.034	0.652

Table S3. Diversity characteristics of individual microsatellites in Germany subpopulation

GER	N	A	Ar	H_o	H_e	HWE	F_{is}	PIC
**AHT4**	17	6	4.00	0.88	0.78	1.000	-0.136	0.741
**AHT5**	17	6	3.17	0.65	0.55	1.000	-0.172	0.527
**HMS1**	17	4	2.74	0.65	0.60	1.000	-0.075	0.520
**HMS2**	17	5	3.33	0.65	0.70	0.119	0.070	0.643
**HMS3**	17	6	3.61	0.65	0.73	0.294	0.118	0.690
**HMS6**	17	4	2.93	0.71	0.63	0.364	-0.118	0.567
**HMS7**	17	5	3.18	0.59	0.69	0.477	0.144	0.628
**HTG4**	17	5	2.82	0.65	0.58	0.031	-0.120	0.508
**HTG6**	17	3	1.44	0.12	0.11	1.000	-0.046	0.109
**HTG7**	17	5	3.40	0.71	0.67	0.425	-0.057	0.608
**HTG10**	17	6	4.10	0.76	0.79	0.999	0.026	0.755
**VHL20**	17	8	4.70	0.82	0.83	1.000	0.010	0.812
**ASB2**	17	5	3.52	0.65	0.74	0.576	0.128	0.698
**ASB17**	10	6	2.93	0.70	0.79	0.315	0.114	0.759
**ASB23**	10	6	3.17	1.00	0.77	0.075	-0.299	0.708
**CA425**	10	5	2.74	0.80	0.73	0.295	-0.103	0.674

Table S4. Diversity characteristics of individual microsatellites in Italian subpopulation

ITA	N	A	Ar	H_o	H_e	HWE	F_{is}	PIC
**AHT4**	4	4	3.27	0.75	0.66	0.330	-0.143	0.605
**AHT5**	4	5	3.96	1.00	0.75	0.000	-0.333	0.671
**HMS1**	4	2	1.93	0.50	0.38	1.000	-0.333	0.305
**HMS2**	4	5	3.87	0.75	0.75	0.000	0.000	0.712
**HMS3**	4	4	3.28	0.75	0.66	0.326	-0.143	0.605
**HMS6**	4	4	3.26	0.75	0.66	0.339	-0.143	0.536
**HMS7**	4	4	3.01	0.50	0.66	0.322	0.238	0.536
**HTG4**	4	3	2.92	1.00	0.66	1.000	-0.524	0.582
**HTG6**	4	1	1.00	0.00	0.00	-	-	0.000
**HTG7**	4	2	2.00	1.00	0.50	0.318	-1.000	0.375
**HTG10**	4	6	4.32	0.75	0.78	0.000	0.040	0.754
**VHL20**	4	6	4.29	0.75	0.81	0.000	0.0767	0.786
**ASB2**	4	5	3.92	1.00	0.75	0.000	-0.333	0.671
**ASB17**	4	5	3.64	0.50	0.75	0.000	0.333	0.712
**ASB23**	4	4	3.26	0.75	0.66	0.000	-0.143	0.346
**CA425**	4	5	3.89	0.75	0.78	1.000	0.040	0.671

Table S5. Diversity characteristics of individual microsatellites in Slovak subpopulation

SVK	N	A	Ar	H_o	H_e	HWE	F_{is}	PIC
**AHT4**	4	2	2.00	0.50	0.5	1.000	0.000	0.375
**AHT5**	4	3	2.35	0.50	0.41	1.000	-0.231	0.371
**HMS1**	4	2	1.93	0.50	0.38	1.000	-0.333	0.305
**HMS2**	4	4	3.28	0.75	0.66	0.359	-0.143	0.605
**HMS3**	4	4	3.03	0.75	0.56	1.000	-0.333	0.524
**HMS6**	4	2	1.93	0.25	0.47	0.335	0.467	0.359
**HMS7**	4	4	3.53	1.00	0.72	1.000	-0.391	0.593
**HTG4**	4	3	2.66	0.75	0.59	0.313	-0.263	0.346
**HTG6**	4	2	1.67	0.25	0.22	1.000	-0.143	0.000
**HTG7**	4	3	2.93	1.00	0.66	1.000	-0.524	0.581
**HTG10**	3	4	3.00	0.67	0.67	0.000	0.000	0.305
**VHL20**	4	4	3.26	0.75	0.66	0.000	-0.143	0.605
**ASB2**	4	4	3.29	1.00	0.66	0.311	-0.524	0.605
**ASB17**	4	5	4.16	1.00	0.78	1.000	-0.280	0.746
**ASB23**	4	4	3.54	1.00	0.72	1.000	-0.391	0.593
**CA425**	4	3	2.88	0.75	0.62	1.000	-0.200	0.555
